# The Service at Westminster Abbey

**Published:** 1917-05-19

**Authors:** 


### THE SERVICE AT WESTMINSTER ABBEY.

The Service of Thanksgiving at Westminster Abbey for the work of the Red Cross during the war comes at the appropriate time when those who know have reason to believe that there is now no theatre of the war in which the work falls below the general level. In a service of this kind it is important to remember that the aim in view is rather to render thanks from those who have been privileged to offer their best in a great work than to signalise a triumph. The point may be mentioned since there is always a temptation on occasions of the kind to disguise a superficial feeling of self-laudation with solemn trappings, but thoughtful people repudiate such temptations with genuine distaste and remember that Kipling's "Lest we forget" applies not only to the occasion for which it was written, but to all solemn thanksgivings whatsoever. It is no superstition which leads men to feel ashamed when the cry "All is well with us" is triumphantly raised around them.

To those capable of rising above the mere surge of pugnacity which every war creates in abundant measure, Red Cross work must appeal as no other can, at least not so profoundly. For it is at heart a recognition that the painful, slow reconstructive work of life, which the treatment of disease best typifies, is the real measure of civilisation, that the triumphs of peace are the real victories, and that the moment of honour is not the moment apparent to the crowd. Such thoughts doubtless moved Bishop Ryle, the Dean of Westminster, to suggest the holding of a solemn service of thanksgiving, which sets, as it were, the Cross of Christ above the eagles of battle, and reminds us that battles, and even the heroism of the field, are transitory, while the peaceful victories and discipline of medicine, hospital life, in a word of the moral forces of man, are permanent, and alone give lasting blessings to us.

It is a startling reminder of this when we recall that while there is not a family in the country which does not owe some personal debt of gratitude to the devotion of Red Cross workers, there are few who will experience any such personal feeling at this or that successful battle. The Service of Thanksgiving for the work of the Red Cross, then, is the expression of the thanks of those who have served and those who have directly or indirectly benefited by the devotion of the former. This is indeed a subject for thanksgiving, and, freed from the suggestion that the organisation and not the work is the subject of honour, our full sympathies are evoked on its behalf. At the same time, "Lest we forget," it should be added that there is still room for improvement, and we hope that one of the effects of the service in the Abbey will be to make all who attend determined to make every Red Cross hospital attain the standard set by the best, and to improve that standard where, as in the case of overworked probationers, for instance, it still lags behind the attainable.

